# A microbial knowledge graph-based deep learning model for predicting candidate microbes for target hosts

**DOI:** 10.1093/bib/bbae119

**Published:** 2024-03-28

**Authors:** Jie Pan, Zhen Zhang, Ying Li, Jiaoyang Yu, Zhuhong You, Chenyu Li, Shixu Wang, Minghui Zhu, Fengzhi Ren, Xuexia Zhang, Yanmei Sun, Shiwei Wang

**Affiliations:** Key Laboratory of Resources Biology and Biotechnology in Western China, Ministry of Education, Provincial Key Laboratory of Biotechnology of Shaanxi Province, College of Life Sciences, Northwest University, Xi’an 710069, China; Key Laboratory of Resources Biology and Biotechnology in Western China, Ministry of Education, Provincial Key Laboratory of Biotechnology of Shaanxi Province, College of Life Sciences, Northwest University, Xi’an 710069, China; Key Laboratory of Resources Biology and Biotechnology in Western China, Ministry of Education, Provincial Key Laboratory of Biotechnology of Shaanxi Province, College of Life Sciences, Northwest University, Xi’an 710069, China; Key Laboratory of Resources Biology and Biotechnology in Western China, Ministry of Education, Provincial Key Laboratory of Biotechnology of Shaanxi Province, College of Life Sciences, Northwest University, Xi’an 710069, China; School of Computer Science, Northwestern Polytechnical University, Xi’an 710129, China; Key Laboratory of Resources Biology and Biotechnology in Western China, Ministry of Education, Provincial Key Laboratory of Biotechnology of Shaanxi Province, College of Life Sciences, Northwest University, Xi’an 710069, China; Key Laboratory of Resources Biology and Biotechnology in Western China, Ministry of Education, Provincial Key Laboratory of Biotechnology of Shaanxi Province, College of Life Sciences, Northwest University, Xi’an 710069, China; Key Laboratory of Resources Biology and Biotechnology in Western China, Ministry of Education, Provincial Key Laboratory of Biotechnology of Shaanxi Province, College of Life Sciences, Northwest University, Xi’an 710069, China; North China Pharmaceutical Group, Shijiazhuang 050015, Hebei, China; National Microbial Medicine Engineering & Research Center, Shijiazhuang 050015, Hebei, China; North China Pharmaceutical Group, Shijiazhuang 050015, Hebei, China; National Microbial Medicine Engineering & Research Center, Shijiazhuang 050015, Hebei, China; Key Laboratory of Resources Biology and Biotechnology in Western China, Ministry of Education, Provincial Key Laboratory of Biotechnology of Shaanxi Province, College of Life Sciences, Northwest University, Xi’an 710069, China; Key Laboratory of Resources Biology and Biotechnology in Western China, Ministry of Education, Provincial Key Laboratory of Biotechnology of Shaanxi Province, College of Life Sciences, Northwest University, Xi’an 710069, China

**Keywords:** knowledge graph (KG), deep learning, bioinformatics, microbe-host interaction (MHI), heterogeneous microbial network (HMN)

## Abstract

Predicting interactions between microbes and hosts plays critical roles in microbiome population genetics and microbial ecology and evolution. How to systematically characterize the sophisticated mechanisms and signal interplay between microbes and hosts is a significant challenge for global health risks. Identifying microbe-host interactions (MHIs) can not only provide helpful insights into their fundamental regulatory mechanisms, but also facilitate the development of targeted therapies for microbial infections. In recent years, computational methods have become an appealing alternative due to the high risk and cost of wet-lab experiments. Therefore, in this study, we utilized rich microbial metagenomic information to construct a novel heterogeneous microbial network (HMN)-based model named KGVHI to predict candidate microbes for target hosts. Specifically, KGVHI first built a HMN by integrating human proteins, viruses and pathogenic bacteria with their biological attributes. Then KGVHI adopted a knowledge graph embedding strategy to capture the global topological structure information of the whole network. A natural language processing algorithm is used to extract the local biological attribute information from the nodes in HMN. Finally, we combined the local and global information and fed it into a blended deep neural network (DNN) for training and prediction. Compared to state-of-the-art methods, the comprehensive experimental results show that our model can obtain excellent results on the corresponding three MHI datasets. Furthermore, we also conducted two pathogenic bacteria case studies to further indicate that KGVHI has excellent predictive capabilities for potential MHI pairs.

## INTRODUCTION

The main precipitants of infectious diseases are the emergence and re-emergence of pathogens, which presents major challenges to public health worldwide [1]. In the last decade, with the rapid evolution of deep sequencing analysis techniques and the rich information of dense and diverse microbial communities, the microbiome has attracted substantial attention in the fields of human health and biological application [2]. Inter-species interactions of proteins [3], DNA and RNA [4] form a complex web, and it can help pathogens disrupt target cellular pathways and gene functions [5]. Therefore, studies of microbe-host interactions (MHIs) can help to understand the molecular mechanisms underlying infection diseases and develop novel biotherapeutics. For example, many studies have found that pathogens typically interact with the protein bottlenecks (proteins at the central locations of important pathways) and hubs (proteins with a lot of interaction partners) in complex spatiotemporally distributed microbe-host protein–protein interaction (PPI) networks [6]. In addition, several large-scale genomics programs have been launched, such as Metagenomics of the Human Intestinal Tract [7] and Human Microbiome Projects [8]. It can aid our understanding of the biological and medical implications of the human microbiome [9]. However, due to time and cost constraints, the number of experimentally validated MHI pairs is very limited. As new virus diseases have been continued to be identified, there is an urgent need to develop a computation-based method to predict MHI to help recommend candidate target hosts from the microbe proteome [10].

Existing MHI computational prediction methods have mainly depended on the evolutionary information of protein sequence [11–13]. Generally, the approaches for MHI prediction can be classified into three categories: structure-based approaches [14], domain-based approaches [15] and sequence-based approaches [16]. The structure-based methods are not applicable when the 3-dimensional (3D) structures of the target proteins are unknown. Similarly, domain-based methods need information on protein domains, which may not be available for each protein in the massive protein-interacting network. Therefore, sequence-based approaches are the most commonly used methodology. While protein functions have been shown that they can be utilized to predict intra-species (e.g. human or plant) PPI [17, 18], and such protein-specific features exist for some extensively studied bacteria (e.g. *Helicobacter pylori* [19] and *Burkholderia pseudomallei* [20]), these features are rare and costly to obtain.

Over the past decade, owing to the rapid development of computer technology, computational-based models can decrease the treatment costs by providing viable strategies for biological experiments. For example, Tsukiyama *et al.* [21] developed a novel LSTM model named LSTM-PHV to predict human-virus interactions (HVI). With the recent development of transfer learning technologies, Yang *et al.* [22] combined evolutionary sequence profile features with a multi-layer perceptron and a Siamese convolutional neural network (CNN) architecture to predict HVI. Sun *et al.* [23] proposed MMiKG, which collected a lot of knowledge of the microbiota-gut-brain axis and depicted it in the form of a KG. Liu-Wei *et al.* [24] designed a deep learning-based model named DeepViral, which embedded human proteins and infected viruses in a shared space through their interacted phenotypes and functions. DeepViral significantly improves existing sequence-based methods by jointly learning protein sequence and phenotype features for predicting HVI. Lian *et al.* [25] developed a machine learning-based method that performed three conventional sequence-based encoding schemes and two host networks to predict human-*Yersinia pestis* PPI. Despite these satisfactory results, such computational methods still suffer some unavoidable disadvantages. For instance, the topological structure of the full microbial networks is not taken into account.

Benefiting from the wide application of sequencing technology and bioinformatics, MHI prediction is not limited to viruses, but also to other microbes related to various hosts, such as HVI, human-bacteria interaction (HBI), phage-bacteria interaction (PBI) and so on [26]. Recently, mining knowledge from the heterogeneous network has been recognized as a novel insight to improve predictive performance, which has complex data structures with nature high-dimensional spatial properties [27]. Thus, graph-based models [28, 29] can model the microbial network structure and extract complex topological information from a global perspective. To date, a number of network embedding algorithms have been presented, and they can be broadly grouped into three types: neural network-based [30], matrix factorization-based [31] and random walk-based [32]. All of these algorithms have achieved great success in the field of bioinformatics. However, this type of approach focuses only on sparse connections between different molecular nodes and ignores the types of edges and node attributes [33]. To overcome the limitations of these methods, knowledge graph (KG)-based methods [34] have gained increased traction [35]. The KG-based embedding techniques can be roughly divided into two categories: (i) semantic matching models [36] and (ii) translational distance models [37]. Methods of the first type measure this by matching the latent semantics of entities and relations, which are captured by vector space representations. On the other hand, the distance between the two entities is the second type of method used to measure the factual plausibility, such as TransE [38], TransR [39] and TransH [40]. Furthermore, with the increasing popularity of graph neural network (GNN), new methods for KG integration have emerged [41]. These methods leverage the idea of deep learning to capture the semantic relations and higher-order structures of graph networks. Prominent examples of such methods include KGAT [42] and R-GCN [43]. However, only a few models have been exploited for predicting MHI.

In this study, we develop a novel KG-based deep learning framework called KGVHI to predict potential interactions between microbes and target hosts by aggregating local biological attribute information and global topological structure information of the microbe-MHI network. In particular, we construct a heterogeneous microbial network (HMN) that includes the human virus, bacteria and phages, and apply three distinct modules to extract meaningful geometric information from the HMN. First, the KG module is applied to obtain the global topological structure information of the entities and edges from HMN. Second, the natural language processing module is adopted to extract the local biological attribute information. Third, we designed a blended deep neural network (DNN) module for integrating these information, training and prediction. The experimental results of 5-fold cross-validation (CV) on three different MHI datasets demonstrate that KGVHI can achieve superior performance, which is superior to state-of-the-art methods. In addition, case studies on two pathogenic bacteria further demonstrated the effectiveness of KGVHI in identifying microbe-related hosts.

## METHODS AND MATERIALS

### Overview of KGVHI model

In this paper, we present a KG-based computational model named KGHIV, which can improve the prediction performance of various microbes and target hosts. The microbe-host-based interaction graph is a directed graph, where the nodes represent entities and the edges indicate entity types of these biomolecules. Let *d* represents a predefined embedding dimension, and $\varOmega =\left\{\left(h,r,t\right)\right\}$ denotes the triplet facts of microbiome KG. The aim of this step is to project the relation $r\in R$ and entity $h\in E$ in a vector space with dimension *d*, where *R* and *E* represent the sets of relations and the set of entities, respectively. We can further promote link prediction and other downstream analysis tasks with this microbial KG embedding (KGE) representation. In the proposed KG of MHI, the proteins are entities and interactions between different microbes and hosts are relations.

**Figure 1:**
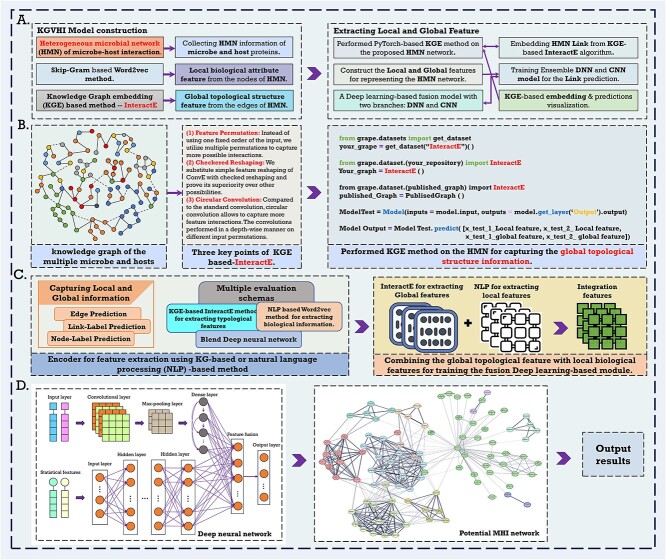
The workflow diagram depicts an overview of the proposed KGVHI model. The heterogeneous microbial network (HMN) consisted of various microbes and target hosts. KGVHI combined the global typological structure feature with the local biological attribute information to infer potential interaction pairs via a blended deep neural network (DNN).

As Figure 1 illustrates, the local biological attribute information of HMN is represented by capturing sequences through natural language processing. Then, KGVHI applied the KGE-based InteractE [44] algorithm to extract the global typological structure features from HMN. As a novel KGE-based method, InteractE has three key ideas to increase the interactions between relationships and entity embeddings. Significantly, we developed a new prediction model named KGVHI and constructed microbes and hosts related to KG to learn protein biological information and generate the topology-preserving representation of numerous microbial interaction networks.

### Benchmark dataset description

#### Human-virus interaction dataset

In this study, we separated the whole MHI dataset into three categories: HVI, HBI and PBI. The HVI datasets were collected from the host-pathogen interaction database (HPIDB) [45]. To better train the proposed model, we first pretreated the collected data set. Specifically, we removed the HVI pairs with a mutual information (MI) score below 0.3 to ensure that all utilization data had high confidence. The MI score represents the confidence level of HVI pairs, which is assigned by InAct [46] and VirHostNet [47]. Then, we used the CD-HIT algorithm with a threshold of 0.95 to remove the redundant pairs [48]. Third, we only selected proteins with lengths longer than 30 and shorter than 1000 residues, which were all composed of standard amino acids. Eventually, the final positive sample is composed by 22 383 HVI pairs from 996 virus proteins and 5882 human proteins.

It is well known that the construction of negative samples does not follow recognized gold standard procedures. A lot of previous works applied the random selection strategy to construct the negative data. However, the random sampling strategy has the risk of assigning positive data to the negative samples [49]. To compensate for this issue, we employed a dissimilarity-negative sampling technique to select the protein pairs that do not have interactions. In detail, the Needleman–Wunsch algorithm of BLOSUM30 [50] was applied to compute the sequence similarities of all virus proteins and assign them a similarity vector. Then, we excluded more than half of total viral proteins with sequence similarity below *Ts* as outliers, which can be calculates as follows:


(1) \begin{equation*} T{\mathrm{s}}_i=f{q}_i-1.5\times i{r}_i \end{equation*}

where $f{q}_i$ represents the first quartile, and the interquartile range of the similarity scores of the *i*th virus protein ${V}_i$ is represented as $i{r}_i$.

The human proteins were retrieved from the UniProKB/Swiss-Prot [51] database with sequences longer than 30 and shorter than 1000. Then, taking into account that human proteins may interact with virus proteins, we removed the proteins whose distance was less than threshold *T*. Based on previous research [52], we set the threshold *T* to 0.8, and the negative samples were constructed by the remaining pairs. Finally, we randomly selected 22 383 negative samples from these candidates.

#### Human-bacteria interaction dataset

The prediction of human and target bacterial pathogens is an important step in systematically analyzing the basic mechanisms of bacterial infection. Thus, we also collected the HBI dataset to further indicate the generalizability of the proposed KGVHI model. The pathogenic bacteria that we collected in HMN were mainly contained of *Yersinia*, *Bacillus* and *Francisella tularensis*. The HBI dataset was collected from the HPIDB database [45]. After pre-trained the collected dataset, we finally collected 8653 HBI pairs, which contain 3502 human proteins and 2563 bacterial proteins.

#### Phage-bacteria interaction dataset

The third MHI dataset that we used in this work is PBI. Due to the abuse of antibiotics, bacterial resistance continues to increase. Bacteriophages (phages) are viruses that specifically infect and lyse bacterial cells. Thus, phage therapy is a potential solution for these questions. The identification of PBI can help people predict phages for target bacteria. The bacteria that we explored in this work are associated with the ESKAPE pathogen, which frequently causes bacterial infections due to their multidrug resistance and aggressive phenotypes. We collected the corresponding datasets from MillardLab [53] and UniProt database [54], which provided 959 phage tail proteins and 522 bacteria receptor-binding proteins.

### Capturing global topological structure information from HMN

The graph embeddings algorithm can mine the hidden information and the linear patterns between edges and entities. Nevertheless, traditional graph embedding methods are not suitable for our work because they focus only on the connection between biological nodes and ignore a large number of edge and entity attributes. In this part, we will focus on utilizing a KG-based algorithm, InteractE, to extract global topological structure information from HMN. Benefiting from the multilayer advantages of CNN, InteractE can increase expressive power while remaining parameter-efficient. Some methods have proved that the expression ability of a model will be improved by adding the possible interaction between embeddings. InteractE extends this concept and leverages three ideas (feature permutation, checkered reshaping and circular convolution) to mine the interactions between the entity and relation feature. In this way, each biological node can be expressed as a specific embedding vector.

**Figure 2:**
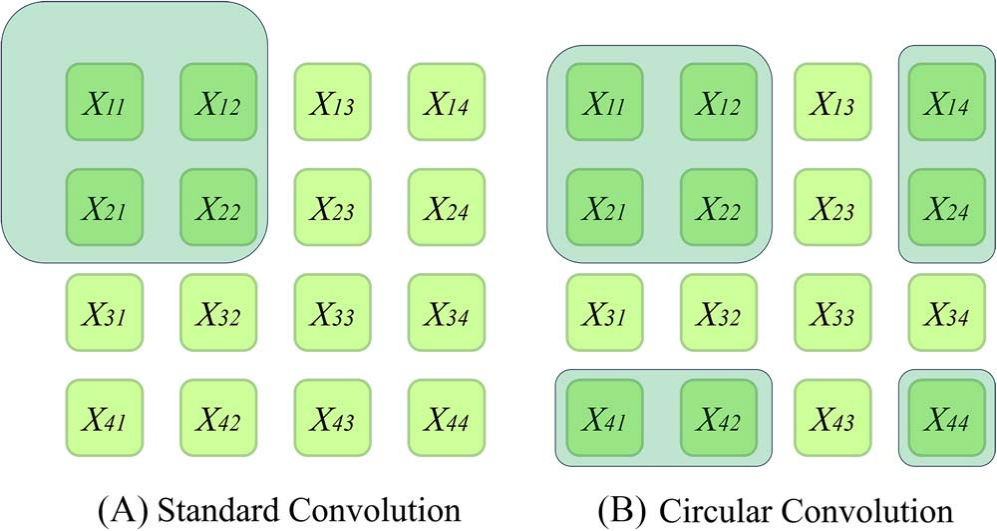
The reason is that circular convolution can capture more information than standard convolution. *X* represents the input matrix, and the location of the filter application is characterized by the shaded area.

Suppose ${e}_s=\left({a}_1,\dots, {a}_d\right),{e}_r=\left({b}_1,\dots, {b}_d\right)$, where ${a}_i$ represents the entity, ${b}_i$ indicates the relation embedding and ${a}_i,{b}_i\in R{\forall}_i$. Let $\phi :{R}^d\times{R}^d\to{R}^{m\times n}$ denotes the reshaping function, which can transform the embeddings into a matrix $\phi :\left({e}_s,{e}_r\right)$, where $m\times n=2d$. Instead of the one fixed order of the input, InteractE adopt multiple permutations to capture more interactions information. First, it proposed ${P}_t=\left[\left({e_s}^1,{e}_s^1\right);\dots; \left({e}_s^t,{e}_s^t\right)\right]$, which represents the *t*-times random permutation of ${e}_r$ and ${e}_s$. In this work, we chose the ${\phi}_{chk}$ as the reshaping operation, where ${\phi}_{chk}\left({e}_s^i,{e}_r^i\right),{\forall}_i\in \left\{1,\dots, t\right\}$ and also defined $\phi \left({P}_t\right)=\left[\phi \left({e}_s^1,{e}_r^1\right);\dots; \phi \left({e}_s^t,{e}_r^t\right)\right]$. From which we can extract complex topological information between entities and relations. In addition, circular convolution was also performed in InteractE algorithm to further increase the possible interactions [55]. Assume that $I\in{R}^{m\times n}$ represents a 2D input with a filter $\omega \in{R}^{k\times k}$, the circular convolution can be calculated as follows:


(2) \begin{equation*} {\left[I\cdotp \omega \right]}_{p,q}=\sum \limits_{i=-\left\lfloor \frac{k}{2}\right\rfloor}^{\left\lfloor \frac{k}{2}\right\rfloor}\sum \limits_{j=-\left\lfloor \frac{k}{2}\right\rfloor}^{\left\lfloor \frac{k}{2}\right\rfloor }{I}_{\left[p-i\right]m,\left[q-j\right]n}{w}_{i,j} \end{equation*}

where $\left\lfloor \cdotp \right\rfloor$ represents the floor function and *n* represents the modulo of *x*. Compared with the standard convolution, the reason that the circular convolution can capture more structure information is shown in Figure 2. By sharing filters across channels, InteractE can perform circular convolution on the input instances in a depth-wise manner, from which we can find the optimal kernel weight [56]. Thus, the InteractE algorithm will flatten these output values and encode them as a feature vector to map into an embedding space $\left({R}^d\right)$. The formulation of the score function is shown as:


(3) \begin{equation*} \psi \left(s,r,o\right)=h\left( vec\left(m\left(\phi \left({P}_k\right)\ast \omega \right)\right)W\right){e}_o \end{equation*}

where vector concatenation, entity embedding matrix, depth-wise circular convolution and learnable weight matrix are represented as $vec\left(\cdotp \right)$, ${e}_o$, $\ast$ and *W*, respectively. In addition, *h* represents *sigmoid* and *m* represents *ReLU*, which were the activated functions during the calculations. In order to obtain a better prediction effect, InteractE also smooths the target label and adopts a standard binary cross-entropy.

### Method of local biological attribute feature embedding from HPI network

In the field of computational biology, natural language processing-based approaches such as doc2vec [57] and word2vec [58] are performed to extract biological attribute information from protein sequences or structures. The weights of the neural network in word2vec can represent and encode different linguistic regularities and patterns of original biological sequences. The word2vec algorithm has two ways (Skip-Gram and CBOW) to learn and extract semantic information from biological sequences. According to a previous study [59], CBOW-based model applies the current words to predict the context, which can learn faster than Skip-Gram, while the second method uses the nearest context to predict the current words for a more accurate output.

In this work, we used the Skip-Gram based word2vec module for capturing the local biological attribute information from HMN. We used the idea of *k*-mers to construct words from each microbial sequence, and the sequences were regarded as sentences. Taking the sequence MTDTLDLE as an example, the units of 4-mers are MTDT, TDTL, DTLD, TLDL and LDLE. We used the genome of Python package to perform the Skip-Gram algorithm for learning the appearance pattern of microbial sequences [60]. We set *k* to 4 and iterated 1000 times to gain a better prediction model [61]. Finally, the word2vec model can produce 64-dimensional vectors in each k-mer to represent the local biological attribute information.

### A deep learning-based classifier by combining CNN and DNN

**Figure 3:**
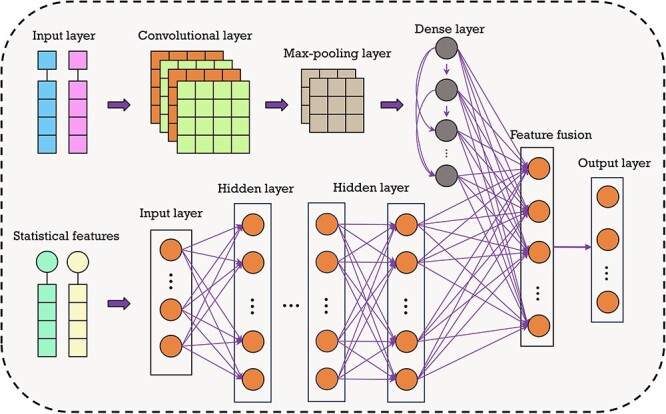
The architecture of the DNN module in the proposed KGVHI model.

To provide a comprehensive prediction model, we propose a blended DNN, which combines CNN with a fully connected neural network (FCNN) to explore multiple pieces of information. The multi-level network is shown in Figure 3. The CNN part of KGVHI is composed of two 2D convolutional layers (CLs), with a kernel size of $4\times 64$, and the max-pooling layers are performed over a $1\times 1$ window. For better training and prediction, all the CLs are equipped with ReLU activations, and the max-pooling layers are performed in a 1 × 1 window [62]. The calculation of the ReLU was performed as follows:


(4) \begin{equation*} {y}_i=\left\{\begin{array}{l}{y}_{a.i}\kern0.5em \mathrm{for}\kern0.5em {y}_{a,i}\ge 0\\{}0\kern1.25em \mathrm{for}\kern0.5em {y}_{a,i}\le 0\end{array}\right. \end{equation*}

where ${y}_{a,i}$ and ${y}_i$ represent the input and output of the ReLU function, respectively. In addition, to prevent overfitting, the dropout technique with a probability of 0.3 was also performed in the DNN module.

The second module is an FCNN to process the global and local information and yield the final prediction results. This module first accepts the input features and then connects them to three layers with 32, 64 and 128 neurons. These two modules flatten and concatenate the features and feed them into a dense layer, where the final softmax function generates the score for the final prediction. In addition, we set the learning rate to 1e-2 and used the AdaGrad optimizer to update the parameters. Finally, the binary cross-entropy function was used as the loss function, and it can be defined as:


(5) \begin{equation*} Loss=-\frac{1}{N}\sum \limits_{i=1}^N{y}_i\cdot \log \left(p\left({y}_i\right)\right)+\left(1-{y}_i\right)\cdot \log \left(1-p\left({y}_i\right)\right) \end{equation*}

where *y* represents the binary class label, and the probability that the results belong to the label *y* is represented as $p(y)$.

## EXPERIMENTS AND RESULTS

### Prediction measures

To fully validate the prediction performance of the proposed KGVHI model, eight commonly used evaluation index were performed, including accuracy (Acc), sensitivity (Sen or Recall), precision (Prec), Matthews correlation coefficient (MCC) and F1-score (F1). These measures can be calculated by:


(6) \begin{equation*} Acc=\frac{TN+ TP}{TN+ TP+ FP+ FN} \end{equation*}

(7) \begin{equation*} Sen= Recall=\frac{TP}{TP+ FN} \end{equation*}

(8) \begin{equation*} Prec=\frac{TP}{FP+ TP} \end{equation*}

(9) \begin{equation*} MCC=\frac{TN\times TP- FN\times FP}{\sqrt{\left( FN+ TN\right)\times \left( TP+ FP\right)\times \left( TN+ FP\right)\times \left( TP+ FN\right)}} \end{equation*}

(10) \begin{equation*} F1=2\times \frac{Prec\times Recall}{Prec+ Recall} \end{equation*}

where true positive (TP) represents the positive samples that were identified as positive, true negative (TN) denotes the negative samples that were predicted to be negative, false positive (FP) represents the negative samples that were predicted to be positive and false negative (FN) denotes the positive samples that were predicted to be negative, respectively. Besides, the receiver operating characteristic (ROC), area under the ROC curves (AUPR) and area under the precision-recall curves (AUPR) are also employed to plot the visualization of the prediction performance of the KGVHI model.

### Evaluation of prediction performance

To fully verify the predictive ability of KGVHI, we performed it on three different microbe-MHIs datasets under a 5-fold CV framework, including HVI, HBI and PBI datasets. First, the whole MHI dataset was split into five equally-sized subsets. Second, four of them were used to train the model, and the remaining one was applied to test the model. This procedure was repeated five times until all subsets were used for validation once.

**Figure 4:**
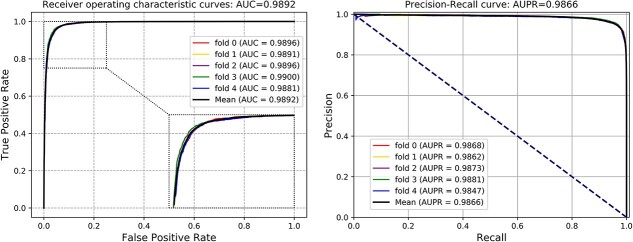
ROC curves obtained in human-virus interaction dataset.

**Figure 5:**
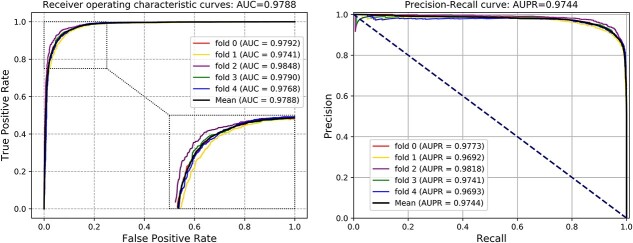
ROC curves obtained in the human-bacteria interaction dataset.

**Figure 6:**
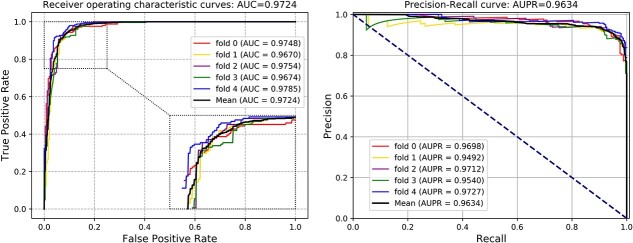
ROC curves obtained in the phage-bacteria interaction dataset.

As illustrated in Table 1, the average Acc values that KGVHI yielded in three MHI datasets (HVI, HBI and PBI) are 95.46%, 92.88% and 92.31%, with SDs of 0.22%, 0.72% and 1.15%, respectively. Besides, KGVHI obtains average AUC values of 0.9892, 0.9788 and 0.9724, with SDs of 0.0007, 0.0039, and 0.0052, respectively, which are shown in Figures 4–6. Moreover, the average AUPR values are 0.9866, 0.9744 and 0.9634, with SDs of 0.0013, 0.0048 and 0.0098, respectively. After analyzing these results, we found that KGVHI obtained the highest Acc value in the HVI dataset, which is over 95%, while the accuracy on the HPI and PBI datasets is relatively low. We attribute this phenomenon to the size of the dataset. In general, the larger the dataset, the more effective the prediction model. Another possibility for this difference is that KGVHI is more sensitive to human and virus proteins. However, we still obtained a high Acc of 0.9231 on the smallest PBI dataset. Collectively, the proposed KGVHI model is efficient and robust in predicting different types of MHI.

**Table 1:** Five-fold CV results on three MHI datasets through KGVHI model

Dataset	Acc. (%)	Sen. (%)	Prec. (%)	MCC (%)	F1 (%)	AUC	AUPR
HVI	95.46 ± 0.22	96.05 ± 0.96	94.95 ± 0.85	90.95 ± 0.43	95.49 ± 0.22	0.9892 ± 0.0007	0.9866 ± 0.0013
HBI	92.88 ± 0.72	90.57 ± 1.66	91.01 ± 1.39	85.86 ± 1.40	93.04 ± 0.67	0.9788 ± 0.0039	0.9744 ± 0.0048
PBI	92.31 + 1.15	88.50 ± 2.02	89.34 ± 1.62	84.89 ± 2.30	92.59 ± 1.11	0.9724 + 0.0052	0.9634 ± 0.0098

**Figure 7:**
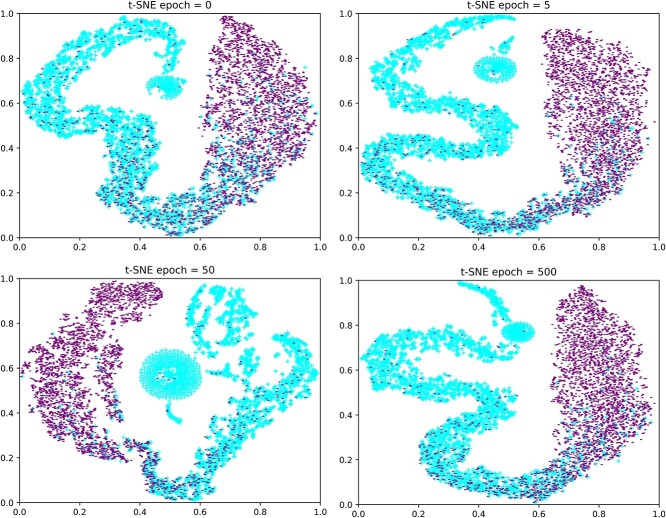
The *t*-SNE transformed 2D visualization of positive and negative samples during different epochs of the proposed KGVHI model.

In order to gain a deeper insight into the distribution of the prediction results, we employ the *t*-distributed stochastic neighbor embedding (*t*-SNE) algorithm to visualize the training process. As shown in Figure 7, the positive samples were indicated by the aqua color ‘+’ and the negative samples were represented by the purple color ‘−’. By comparison, one can see that when the training epochs increased, KGVHI could roughly isolate and distinguish these biological samples.

### Best embedding dimensions of KGVHI model

**Figure 8:**
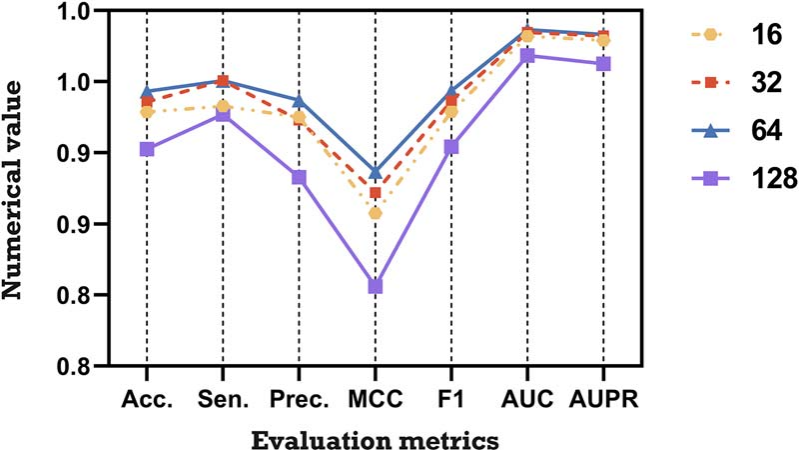
Prediction performance of KGVHI with a 5-fold CV framework of different embedding dimensions.

The embedding dimension is a hyperparameter for representation learning that can encode the topological structure of HMN into a specific vector space. It is a challenge but necessary for KGVHI to choose an appropriate embedding dimension that can be applied to multiple prediction tasks. The chosen dimension must be large enough to be effective for modeling, but small enough to be computationally efficient. In this section, we designed an experiment to find the optimal embedding dimensions for the purpose of better model performance. Specifically, we applied the same embedding method to the MHI dataset with different embedding dimensions (16, 32, 64 and 128) to extract local and global features from HMN. Table 2 and Figure 8 list the prediction results that were obtained from these four different dimensions. We found that the prediction accuracy tends to increase and then decrease as the embedding dimension increases. In the proposed KGVHI model, we found that 64-dimension can extract the richest topological information without too much noise data. Finally, it can be concluded that KGVHI has a stabilizing ability to predict potential target hosts for various microorganisms.

**Table 2:** Find the optimal embedding dimensions of the HVI dataset

Fold	ACC (%)	Sen (%)	Prec (%)	MCC (%)	F1 (%)	AUC	AUPR
16	94.29 ± 0.13	94.63 ± 1.32	94.01 ± 1.09	88.60 ± 0.27	94.31 ± 0.16	0.9855 ± 0.0009	0.9832 ± 0.0011
32	94.85 ± 0.33	96.05 ± 1.15	93.84 ± 1.43	89.76 ± 0.63	94.92 ± 0.28	0.9878 ± 0.0010	0.9858 ± 0.0012
64	95.46 ± 0.22	96.05 ± 0.96	94.95 ± 0.85	90.95 ± 0.43	95.49 ± 0.22	0.9892 ± 0.0007	0.9866 ± 0.0013
128	92.20 ± 0.24	94.16 ± 0.97	90.62 ± 1.09	84.48 ± 0.41	92.35 ± 0.17	0.9748 ± 0.0010	0.9701 ± 0.0013

### Compared KGVHI with some state-of-the-art methods

To comprehensively illustrate the validity of the proposed method, several KG-embedding methods are chosen to compare with our method, including ConvE [63], Distmult [64] and TransE [40]. In addition, to demonstrate the effectiveness of the proposed deep learning-based classifier, we compared it to some widely used machine learning classifiers, including support vector machine (SVM; *c* = 0.045, *g* = 1.3), random forest (RF, *n* = 13) and gradient-boosting decision tree (GBDT, *n* = 5).

**Figure 9:**
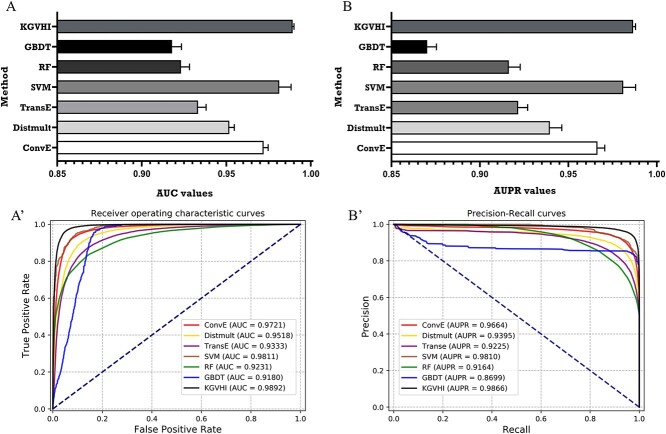
The performance of KGVHI and comparison methods under a 5-fold CV framework on the HVI dataset.

In this part, these comparison experiments were performed on the HVI dataset. We first compared KGVHI with these methods under a 5-fold CV framework, and all the prediction results are listed in Table 3. To ensure a fair and thorough comparison, the global features that were extracted from KG methods are combined with the same local features. Similarly, when comparing the DNN module with these machine learning-based classifiers, we used the same local and global features and the same division dataset. Moreover, the parameters of KGE algorithms were set as default, and the parameters of machine learning classifiers were fine-tuned. In this way, these methods may not achieve perfect predictive performance. Here, however, our goal is to validate the effectiveness of these comparison methods, so we will not seek to obtain little progress by fine-tuning parameters. It can be seen from Figure 9 that KGVHI outperforms all comparison methods in AUC and AUPR values. We attribute this performance to the fact that KGVHI can effectively combine InteractE and the word2vec algorithm to capture latent information of HMN. We also attribute this to the proposed blended DNN, which can fuse the global and local-derived features.

**Table 3:** Comparison results with some state-of-the-art methods on the HVI dataset

Method	ACC (%)	Sen (%)	Prec (%)	MCC (%)	F1 (%)	AUC	AUPR
ConvE	92.11 ± 0.43	93.25 ± 1.16	91.18 ± 1.12	84.25 ± 0.86	92.2 ± 0.42	0.9721 ± 0.0026	0.9664 ± 0.0042
DistMult	88.87 ± 0.70	92.05 ± 2.86	86.7 ± 2.87	78.05 ± 0.94	89.22 ± 0.37	0.9518 ± 0.0029	0.9395 ± 0.0067
TransE	86.42 ± 0.60	88.07 ± 1.66	85.31 ± 1.77	72.93 ± 1.20	86.64 ± 0.50	0.9333 ± 0.0047	0.9225 ± 0.0058
SVM	92.19 ± 2.24	96.11 ± 1.36	89.16 ± 2.73	84.65 ± 4.37	92.5 ± 2.09	0.9811 ± 0.0070	0.9810 ± 0.0069
RF	84.70 ± 0.42	83.13 ± 1.17	85.86 ± 1.44	69.46 ± 0.91	84.46 ± 0.32	0.9231 ± 0.0050	0.9164 ± 0.0064
GBDT	86.67 ± 0.52	99.14 ± 0.48	79.37 ± 0.71	75.75 ± 0.88	88.15 ± 0.4	0.9180 ± 0.0055	0.8699 ± 0.0053
KGVHI	95.46 ± 0.22	96.05 ± 0.96	94.95 ± 0.85	90.95 ± 0.43	95.49 ± 0.22	0.9892 ± 0.0007	0.9866 ± 0.0013

### Ablation experiments

The above comparison results demonstrate the validity of KGVHI, and the robustness of the proposed model is benefited from its design: a KG-based embedding method to generate the global topology feature and a natural language processing-based method to capture local biological attribute features. Here, an ablation study was carried out, and the following variant of KGVHI was considered and performed on the HVI dataset. From which we can explore the contribution of these modules.

(i) KGVHI_G is a variant of KGVHI that only uses the global topological structure features.
(ii) KGVHI_L is a variant of KGVHI that only uses the local biological attribute features.

**Figure 10:**
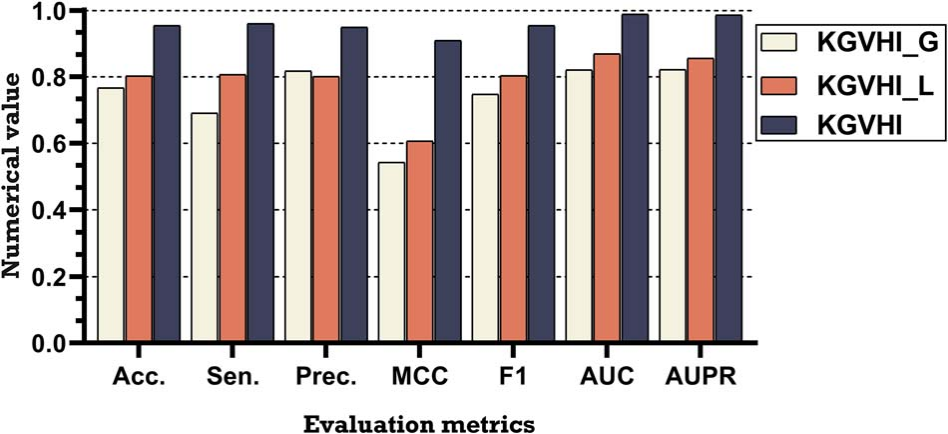
The comparison results of the different variants on the HVI dataset.

The prediction results of KGVHI and its two variants are listed in Table 4. We can see that the prediction power of KGVHI is impaired when any module is deleted, meaning that all components are essential to KGVHI. From Figure 10, we can see that the KGVHI_G variant was affected significantly after removing the local features, with the Acc and MCC values reduced by 18.72% and 36.65%, respectively. Meanwhile, the Acc and MCC values of KGVHI_L were reduced by 15.16% and 30.26%, respectively. From the comparison results of KGVHI and KGVHI_L, we can conclude that the application of global topological attribute information can significantly enhance the prediction ability of the proposed model. Moreover, the comparison results of KGVHI_G and KGVHI_L further demonstrated that biological attribute information is essential for predicting MHI. We also found that removing global information also leads to a decrease in model performance, suggesting that the topological information of HMN must be taken into account.

**Table 4:** The comparison results of KGVHI and different variants yielded on the HVI dataset under 5-fold CV

Model	ACC (%)	Sen (%)	Prec (%)	MCC (%)	F1 (%)	AUC	AUPR
KGVHI_G	76.74 ± 0.97	69.08 ± 4.74	81.83 ± 3.16	54.30 ± 1.93	74.75 ± 1.77	0.8216 ± 0.0099	0.8217 ± 0.0109
KGVHI_L	80.30 ± 0.43	80.73 ± 3.03	80.13 ± 1.77	60.69 ± 0.84	80.37 ± 0.77	0.8699 ± 0.0045	0.8563 ± 0.0074
KGVHI	95.46 ± 0.22	96.05 ± 0.96	94.95 ± 0.85	90.95 ± 0.43	95.49 ± 0.22	0.9892 ± 0.0007	0.9866 ± 0.0013

### Case study

To further verify the predictive ability of KGVHI, we carried out case studies on two common bacteria, *Acinetobacter baumannii* and *Staphylococcus aureus*. *Acinetobacter baumannii*, a gram-negative pathogenic bacterium, is one of the most common cases of nosocomial infection. It usually causes severe bacterial diseases, such as meningitis, pneumonia, endocarditis, peritonitis and urinary tract and skin infections. The widespread use and misuse of antibiotics has led to the development of resistance in *A. baumannii*, which has become multidrug-resistant *A. baumannii* and has attracted more and more attention from academic and microbiology researchers. We also made a case study on *S. aureus*, which is a gram-positive bacterium with strong pathogenicity and is common in clinics. It is always parasitic in the skin, nasal cavity, throat and septic sores of humans and animals, and also ubiquitous in the air, sewage and other environments.

For each bacterial species, phages that have known interactions with the bacteria are first removed. Then, the predicted scores of candidate phages are sorted in descending order according to the KGVHI model. In other words, KGVHI only relies on the known microbiome knowledge and the extracted local and global information of the training sets. Assume a microbiome adjacency matrix A. Then, all the MHI samples will be ranked based on the prediction scores. After predicting, the top 30 ranked samples will be further indicated by public databases and available literature. As can be seen in Tables 5 and 6, 27 and 29 of the top 30 predicted *A. baumannii* and *S. aureus* target phages have been validated by previous studies. These prediction results further demonstrated that our model has generalizability and validity. We hope that our model can help to predict more and more potential MHI pairs.

**Table 5:** Top 30 predicting samples of *Acinetobacter baumannii* related phages

Rank	UniProt id	Score	Evidence	Rank	UniProt id	Score	Evidence
1	ARB06757.1	0.99984	Confirmed	16	A0A068CGF5	0.99701	Confirmed
2	A0A0A0RMQ0	0.99982	Confirmed	17	ARQ94727.1	0.99653	Confirmed
3	A0A2S1GTT1	0.99978	Confirmed	18	A0A221SBN3	0.99647	Confirmed
4	A0A2S1GTS5	0.99976	Confirmed	19	U5PW98	0.99646	Confirmed
5	A0A386KK43	0.99973	Confirmed	20	A0A190XCC0	0.99627	Confirmed
6	A0A172Q097	0.99955	Confirmed	21	A0A386KAA1	0.99595	Confirmed
7	AWD93192.1	0.99943	Confirmed	22	YP_009206147.1	0.99560	NA
8	A0A346FJ10	0.99925	Confirmed	23	APD19509.1	0.99560	NA
9	ARQ94726.1	0.99904	Confirmed	24	A0A0P0IE19	0.99505	Confirmed
10	A0A220NQG3	0.99893	Confirmed	25	A0A075DXN1	0.99494	Confirmed
11	CEK40295.1	0.99780	NA	26	AXY82734.1	0.99469	Confirmed
12	A0A386KM25	0.99768	Confirmed	27	A0A0P0IJ73	0.99466	Confirmed
13	A0A220NQG5	0.99756	Confirmed	28	J7I0X3	0.99464	Confirmed
14	AFV51556.1	0.99725	Confirmed	29	E5KJQ6	0.99421	Confirmed
15	AXY82661.1	0.99722	Confirmed	30	A0A0A0RR02	0.99391	Confirmed

**Table 6:** Top 30 predicting sample of *S. aureus* related phages

Rank	UniProt id	Score	Evidence	Rank	UniProt id	Score	Evidence
1	A0A2D1GPH4	0.99664	Confirmed	16	YP_002332534.1	0.99518	Confirmed
2	A0A2R4P8T8	0.99661	Confirmed	17	A0A1D6Z279	0.99515	Confirmed
3	A0A345AQC0	0.99655	Confirmed	18	A0A0E4BZU6	0.99505	Confirmed
4	Q8SDP3	0.99647	Confirmed	19	YP_009006774.1	0.99469	Confirmed
5	QAY02680.1	0.99638	Confirmed	20	M9NSU7	0.99464	Confirmed
6	AUG85753.1	0.99636	Confirmed	21	YP_001004393.1	0.99456	Confirmed
7	APD20961.1	0.99630	Confirmed	22	A0A220BYL5	0.99446	Confirmed
8	AWD93112.1	0.99619	Confirmed	23	AUS03378.1	0.99443	Confirmed
9	A0A345AQC1	0.99616	Confirmed	24	YP_001004325.1	0.99438	Confirmed
10	APD20962.1	0.99612	Confirmed	25	A3RE05	0.99422	NA
11	A0A2R4P8U9	0.99595	Confirmed	26	A0A2K9V4F5	0.99421	Confirmed
12	YP_009278564.1	0.99587	Confirmed	27	A0A0E3XCZ0	0.99420	Confirmed
13	A0A1D6Z282	0.99566	Confirmed	28	A0A0H3U4S3	0.99420	Confirmed
14	APD20939.1	0.99548	Confirmed	29	YP_009006776.1	0.99419	Confirmed
15	S4V7L6	0.99534	Confirmed	30	D2K045	0.99419	Confirmed

## DISCUSSION AND CONCLUSION

Recent studies have clearly demonstrated that microorganisms play vital roles in their reaction mechanisms. Moreover, predicting microbe-MHIs can benefit people by facilitating drug development, which also helps in the development of novel phage therapies. Compared with traditional wet-lab experiments, it is more convenient to use computational methods for identifying target viruses for existing human proteins or novel phages for known bacteria. However, few of them have been used to address these hard questions, possibly because only a few interaction pairs have been experimentally validated and applied to computational models.

In this paper, we propose a KG-based model named KGVHI to predict potential MHI by combining local biological attribute information with global topological structure information, which has inductive and scalable capabilities. This model not only considers phage and bacterial biochemical information, but also employs the idea of KG to extract global topological structure information from the HMN. Specifically, the word2vec algorithm is used to extract biological features from HMN. It is worth nothing that word2vec can preserve richer semantic information in biological sequence. In recent years, due to the rapid advances in high-throughput technologies, the metagenomic alterations of microbiota have increased quickly. However, there are still a few available data points for predicting host-associated microbes. In such a case, KGVHI performs a KG algorithm, InteractE, to integrate the neighboring features from HMN, thus avoiding the negative impact of the lack of a sufficient dataset. The presentation of multivariate information ensures KGVHI fully realizes the potential ability of HMN, which can increase the prediction accuracy of microbes and target hosts. On the other hand, KGVHI also takes advantage of the powerful mechanism of the blended DNN to accurately conduct the prediction task. The blended DNN has more hidden layers and neurons, which can further increase the ability of KGVHI to process the multivariate heterogeneous information and perform robustly in predicting MHI. In addition, comparison experiments with various machine learning and KG-based methods also demonstrated that the presented method performs well and is applicable to different microbe-host bioinformatics tasks. We also carried out case studies on gram-positive and negative-bacteria to further indicate that KGVHI has practical applications.

Although KGVHI shows surprising predictive performance, many challenges remain for this model. For example, the negative samples are selected by the dissimilarity negative sampling technique, which may cause certain noise to limit predicting ability. Another challenge is that in the MHI, consider three different relationships among microbes, including human-virus, human-bacteria and phage-bacteria. However, there were other possible interactions in MHI besides these three relationships. In this regard, we will try to collect more different types of MHI pairs to construct a more comprehensive HMN, from which KGVHI can capture more expressive biological features. However, as the microorganism network becomes more complex, the redundancy and noise in functional information are becoming more and more severe. Thus, it may cause new difficulties for the prediction models about attribute fusion capabilities. To address this issue, we intend to explore novel KG methods to provide new insight for the downstream prediction task. In the next step, we would try to integrate more microbial attributes into HMN to improve the predictive performance of KGVHI.

**Key Points**

- KGVHI constructed a novel a HMN and used a novel algorithm to construct the negative dataset.
- KGVHI integrated the global topological structure information with local biological attribute information to predict candidate microbes for target hosts.
- Knowledge graph embedding for capturing the global topological structure information from MHN.

## Data Availability

The score code and whole data is available at https://github.com/NWUJiePan/KGVHI

## Notes

**Jie Pan** is a Phd student at Northwest University. His research interests include bioinformatics, microbiology, machine learning, and network analysis.

**Zhen Zhang** is a Phd student at Northwest University. His research intersts include bioinformatics, machine learning, and complex network.

**Ying Li, Jiaoyang Yu, Chenyu Li, Shixu Wang and Minghui Zhu** are master students of Northwest University. Their research interests include phage and bacterial whole-genome data analysis, transcriptome.

**Zhuhong You** is a Professor of Northwestern Polytechnical University. His research interest include data mining, machine learning, computational biology and bioinformatics.

**Xuexia Zhang** is the professor in North China Pharmaceutical Group and National Microbial Medicine Engineering & Research Center. Her research interests include Microbiology and biochemical pharmacy.

**Fengzhi Ren** is the professor in North China Pharmaceutical Group and National Microbial Medicine Engineering & Research Center. Her research interests include microbial genetics and microbial communities.

**Yanmei Sun** is a Professor of Northwest University. Her research interests include host-microbiome interactions and microbial communities.

**Shiwei Wang** is a Professor of Northwest University. His research interests include physiological studies on Pseudomonas aeruginosa and molecular biology of phages and their applications.

## References

[1] Morens DM , Folkers GK, Fauci AS. The challenge of emerging and re-emerging infectious diseases. Nature 2004;430(6996):242–9. 15241422 10.1038/nature02759 PMC7094993

[2] Cho I , Blaser MJ. The human microbiome: at the interface of health and disease. Nat Rev Genet 2012;13(4):260–70. 22411464 10.1038/nrg3182 PMC3418802

[3] Dyer MD , Murali T, Sobral BW. The landscape of human proteins interacting with viruses and other pathogens. PLoS Pathog 2008;4(2):e32. 18282095 10.1371/journal.ppat.0040032 PMC2242834

[4] Fajardo T, Jr, Sung P-Y, Roy P. Disruption of specific RNA-RNA interactions in a double-stranded RNA virus inhibits genome packaging and virus infectivity. PLoS Pathog 2015;11(12):e1005321. 26646790 10.1371/journal.ppat.1005321 PMC4672896

[5] Brodsky IE , Medzhitov R. Targeting of immune signalling networks by bacterial pathogens. Nat Cell Biol 2009;11(5):521–6. 19404331 10.1038/ncb0509-521

[6] Ahmed H , Howton T, Sun Y, et al. Network biology discovers pathogen contact points in host protein-protein interactomes. Nat Commun 2018;9(1):2312. 29899369 10.1038/s41467-018-04632-8 PMC5998135

[7] Ehrlich SD , Consortium M. MetaHIT: The European Union Project on metagenomics of the human intestinal tract. In: Metagenomics of the Human Body, 2011, 307–16.

[8] The Human Microbiome Project Consortium. A framework for human microbiome research. Nature 2012;486(7402):215–21. 22699610 10.1038/nature11209 PMC3377744

[9] Zhao Y , Wang C-C, Chen X. Microbes and complex diseases: from experimental results to computational models. Brief Bioinform 2021;22:bbaa158. 32766753 10.1093/bib/bbaa158

[10] Pan J , You W, Lu X, et al. GSPHI: a novel deep learning model for predicting phage-host interactions via multiple biological information. Comput Struct Biotechnol J 2023;21:3404–13. 37397626 10.1016/j.csbj.2023.06.014 PMC10314231

[11] Mock F , Viehweger A, Barth E, Marz M. VIDHOP, viral host prediction with deep learning. Bioinformatics 2021;37(3):318–25. 32777818 10.1093/bioinformatics/btaa705 PMC7454304

[12] Dey L , Chakraborty S, Mukhopadhyay A. Machine learning techniques for sequence-based prediction of viral–host interactions between SARS-CoV-2 and human proteins. Biom J 2020;43(5):438–50. 10.1016/j.bj.2020.08.003 PMC7470713 33036956

[13] Yang X , Yang S, Li Q, et al. Prediction of human-virus protein-protein interactions through a sequence embedding-based machine learning method. Comput Struct Biotechnol J 2020;18:153–61. 31969974 10.1016/j.csbj.2019.12.005 PMC6961065

[14] Mariano R , Wuchty S. Structure-based prediction of host–pathogen protein interactions. Curr Opin Struct Biol 2017;44:119–24. 28319831 10.1016/j.sbi.2017.02.007

[15] Kataria R , Kaundal R. Deciphering the host–pathogen interactome of the wheat–common bunt system: a step towards enhanced resilience in next generation wheat. Int J Mol Sci 2022;23(5):2589. 35269732 10.3390/ijms23052589 PMC8910311

[16] Matthews LR , Vaglio P, Reboul J, et al. Identification of potential interaction networks using sequence-based searches for conserved protein-protein interactions or “interologs”. Genome Res 2001;11(12):2120–6. 11731503 10.1101/gr.205301 PMC311221

[17] Ray S , Lall S, Bandyopadhyay S. A deep integrated framework for predicting SARS-CoV2–human protein-protein interaction. IEEE Trans Emerg Top Comput 2022;6(6):1463–72.

[18] Pan J , You ZH, Li LP, et al. Dwppi: a deep learning approach for predicting protein–protein interactions in plants based on multi-source information with a large-scale biological network. Front Bioeng Biotechnol 2022;10:807522. 35387292 10.3389/fbioe.2022.807522 PMC8978800

[19] Liu M , Chen H, Gao D, et al. Identification of *Helicobacter pylori* membrane proteins using sequence-based features. Comput Math Methods Med 2022;2022:1–7. 10.1155/2022/7493834 PMC8769816 35069791

[20] Loaiza CD , Duhan N, Lister M, Kaundal R. In silico prediction of host–pathogen protein interactions in melioidosis pathogen *Burkholderia pseudomallei* and human reveals novel virulence factors and their targets. Brief Bioinform 2021;22:bbz162. 32444871 10.1093/bib/bbz162

[21] Tsukiyama S , Hasan MM, Fujii S, Kurata H. LSTM-PHV: prediction of human-virus protein–protein interactions by LSTM with word2vec. Brief Bioinform 2021;22(6):bbab228. 34160596 10.1093/bib/bbab228 PMC8574953

[22] Yang X , Yang S, Lian X, et al. Transfer learning via multi-scale convolutional neural layers for human–virus protein–protein interaction prediction. Bioinformatics 2021;37(24):4771–8. 34273146 10.1093/bioinformatics/btab533 PMC8406877

[23] Sun H , Song Z, Chen Q, et al. MMiKG: a knowledge graph-based platform for path mining of microbiota–mental diseases interactions. Brief Bioinform 2023;24(6):bbad340. 37779250 10.1093/bib/bbad340

[24] Liu-Wei W , Kafkas Ş, Chen J, et al. DeepViral: prediction of novel virus–host interactions from protein sequences and infectious disease phenotypes. Bioinformatics 2021;37(17):2722–9. 33682875 10.1093/bioinformatics/btab147 PMC8428617

[25] Lian X , Yang S, Li H, et al. Machine-learning-based predictor of human–bacteria protein–protein interactions by incorporating comprehensive host-network properties. J Proteome Res 2019;18(5):2195–205. 30983371 10.1021/acs.jproteome.9b00074

[26] Cheng J , Lin Y, Xu L, et al. ViRBase v3. 0: a virus and host ncRNA-associated interaction repository with increased coverage and annotation. Nucleic Acids Res 2022;50(D1):D928–33. 34723320 10.1093/nar/gkab1029 PMC8728225

[27] Liu D , Ma Y, Jiang X, He T. Predicting virus-host association by Kernelized logistic matrix factorization and similarity network fusion. BMC Bioinformatics 2019;20:1–10. 31787095 10.1186/s12859-019-3082-0 PMC6886165

[28] Du H , Chen F, Liu H, Hong P. Network-based virus-host interaction prediction with application to SARS-CoV-2. Patterns 2021;2(5):100242. 33817672 10.1016/j.patter.2021.100242 PMC8006187

[29] Suratanee A , Buaboocha T, Plaimas K. Prediction of human-plasmodium vivax protein associations from heterogeneous network structures based on machine-learning approach. Bioinformatics Biol Insights 2021;15:11779322211013350. 10.1177/11779322211013350 PMC8212370 34188457

[30] Tang J , Qu M, Wang M, et al. Line: Large-scale information network embedding. In: Proceedings of the 24th International Conference on World Wide Web, 2015, pp. 1067–77.

[31] Belkin M , Niyogi P. Laplacian eigenmaps for dimensionality reduction and data representation. Neural Comput 2003;15(6):1373–96.

[32] Perozzi B , Al-Rfou R, Skiena S, Deepwalk: Online learning of social representations. In: Proceedings of the 20th ACM SIGKDD International Conference on Knowledge Discovery and Data Mining, 2014, pp. 701–10.

[33] Nickel M , Murphy K, Tresp V, Gabrilovich E. A review of relational machine learning for knowledge graphs. Proc IEEE 2015;104(1):11–33.

[34] Guan N , Song D, Liao L. Knowledge graph embedding with concepts. Knowledge-Based Systems 2019;164:38–44.

[35] Yang F , Zou Q, Gao B. GutBalance: a server for the human gut microbiome-based disease prediction and biomarker discovery with compositionality addressed. Brief Bioinform 2021;22(5):bbaa436. 33515036 10.1093/bib/bbaa436

[36] Xiong C , Power R, Callan J, Explicit semantic ranking for academic search via knowledge graph embedding. In: Proceedings of the 26th International Conference on World Wide Web, 2017, 1271–9.

[37] Zhang S , Sun Z, Zhang W. Improve the translational distance models for knowledge graph embedding. J Intell Inf Syst 2020;55:445–67.

[38] Bordes A , Usunier N, Garcia-Duran A, et al. Translating embeddings for modeling multi-relational data. Adv Neur Inf Process Syst 2013;26.

[39] Lin Y , Liu Z, Sun M, et al. Learning entity and relation embeddings for knowledge graph completion. In: Proceedings of the AAAI Conference on Artificial Intelligence, 2015, 29(1).

[40] Wang Z , Zhang J, Feng J, Chen Z, Knowledge graph embedding by translating on hyperplanes. In: Proceedings of the AAAI Conference on Artificial Intelligence, 2014, 28(1).

[41] Wang Y , Wu J, Yan J, et al. Comparative genome analysis of plant ascomycete fungal pathogens with different lifestyles reveals distinctive virulence strategies. BMC genomics 2022;23(1):34. 34996360 10.1186/s12864-021-08165-1 PMC8740420

[42] Wang X , He X, Cao Y, et al. KGAT: Knowledge graph attention network for recommendation. In: Proceedings of the 25th ACM SIGKDD International Conference on Knowledge Discovery & Data Mining, 2019, pp. 950–8.

[43] Schlichtkrull M , Kipf T. N., Bloem P, et al. Modeling relational data with graph convolutional networks. In: The Semantic Web: 15th International Conference, ESWC 2018, Heraklion, Crete, Greece, June 3–7, 2018, Proceedings 15, 2018, pp. 593–607: Springer.

[44] Vashishth S , Sanyal S, Nitin V, et al. InteractE: improving convolution-based knowledge graph embeddings by increasing feature interactions. arXiv 2020;34(03):3009–16.

[45] Ammari MG , Gresham CR, McCarthy FM, Nanduri B. HPIDB 2.0: a curated database for host–pathogen interactions. Database 2016;2016:baw103. 27374121 10.1093/database/baw103 PMC4930832

[46] Kerrien S , Aranda B, Breuza L, et al. The IntAct molecular interaction database in 2012. Nucleic Acids Res 2012;40(D1):D841–6. 22121220 10.1093/nar/gkr1088 PMC3245075

[47] Guirimand T , Delmotte S, Navratil V. VirHostNet 2.0: surfing on the web of virus/host molecular interactions data. Nucleic Acids Res 2015;43(D1):D583–7. 25392406 10.1093/nar/gku1121 PMC4383936

[48] Fu L , Niu B, Zhu Z, et al. CD-HIT: accelerated for clustering the next-generation sequencing data. Bioinformatics 2012;28(23):3150–2. 23060610 10.1093/bioinformatics/bts565 PMC3516142

[49] Singh AS , Masuku MB. Sampling techniques and determination of sample size in applied statistics research: an overview. Int J Econ Commer Manage 2014;2(11):1–22.

[50] Likic V . The Needleman-Wunsch algorithm for sequence alignment. In: Lecture given at the 7th Melbourne Bioinformatics Course, Bi021 Molecular Science and Biotechnology Institute. University of Melbourne, 2008, 1–46.

[51] UniProt: the universal protein knowledgebase in 2023. Nucleic Acids Res 2023;51(D1):D523–31. 36408920 10.1093/nar/gkac1052 PMC9825514

[52] Eid F-E , ElHefnawi M, Heath LS. DeNovo: virus-host sequence-based protein–protein interaction prediction. Bioinformatics 2016;32(8):1144–50. 26677965 10.1093/bioinformatics/btv737

[53] Cook R , Brown N, Redgwell T, et al. INfrastructure for a PHAge REference database: identification of large-scale biases in the current collection of cultured phage genomes. Phage 2021;2(4):214–23. 36159887 10.1089/phage.2021.0007 PMC9041510

[54] UniProt Consortium . UniProt: a worldwide hub of protein knowledge. Nucleic Acids Res 2019;47(D1):D506–15. 30395287 10.1093/nar/gky1049 PMC6323992

[55] Wang T-H , Huang H-J, Lin J-T, et al. Omnidirectional CNN for visual place recognition and navigation. In: 2018 IEEE International Conference on Robotics and Automation (ICRA), 2018, pp. 2341–8: IEEE.

[56] Chollet F , Xception: Deep learning with depthwise separable convolutions. In: Proceedings of the IEEE Conference on Computer Vision and Pattern Recognition, 2017, pp. 1251–8.

[57] Lau JH , Baldwin T. An empirical evaluation of doc2vec with practical insights into document embedding generation. arXiv:1607.05368, 2016.

[58] Goldberg Y , Levy O. word2vec explained: deriving Mikolov et al.’s negative-sampling word-embedding method. arXiv:1402.3722, 2014.

[59] İrsoy O , Benton A, Stratos K. Corrected CBOW performs as well as skip-gram. arXiv:2012.15332, 2020.

[60] Řehůřek R , Sojka P. Software framework for topic modelling with large corpora. 2010.

[61] Cao Z , Pan X, Yang Y, et al. The lncLocator: a subcellular localization predictor for long non-coding RNAs based on a stacked ensemble classifier. Bioinformatics 2018;34(13):2185–94. 29462250 10.1093/bioinformatics/bty085

[62] Nair V , Hinton GE, Rectified linear units improve restricted Boltzmann machines. In: Proceedings of the 27th International Conference on Machine Learning (ICML-10), 2010, pp. 807–14.

[63] Dettmers T , Minervini P, Stenetorp P, Riedel S, Convolutional 2d knowledge graph embeddings. In: Proceedings of the AAAI Conference on Artificial Intelligence, 2018, vol. 32( 1).

[64] Tran HN , Takasu A. Analyzing knowledge graph embedding methods from a multi-embedding interaction perspective. arXiv:1903.11406, 2019.

